# Trazodone-Induced Ischemic Priapism Successfully Managed With Conservative Therapy: A Case Report

**DOI:** 10.7759/cureus.88603

**Published:** 2025-07-23

**Authors:** Stanislaw Szymkiewicz

**Affiliations:** 1 Department of Urology, Janusz Korczak Provincial Specialist Hospital, Slupsk, POL

**Keywords:** conservative management, drug-induced priapism, ischemic priapism, trazodone, urological emergency

## Abstract

Ischemic priapism is a urological emergency requiring prompt diagnosis and intervention to prevent permanent erectile dysfunction. We present the case of a 29-year-old male admitted for a painful erection lasting 10 hours. The patient had no history of trauma, hematological disease, or use of erectile medications. He had recently started trazodone (Trittico) for insomnia. Clinical examination and Doppler ultrasound confirmed low-flow priapism. Cavernosal blood gas analysis revealed severe hypoxia and acidosis. Conservative management including aspiration, irrigation, intracavernosal ephedrine, anticoagulation, and ice application led to complete detumescence without surgical intervention. The case highlights trazodone as a potential cause of ischemic priapism and the importance of early, structured management.

## Introduction

Ischemic priapism is a rare urological emergency characterized by a persistent, painful penile erection that lasts beyond four hours and is unrelated to sexual stimulation. If not promptly diagnosed and treated, it can lead to irreversible corporal fibrosis and permanent erectile dysfunction [1,2]. The overall incidence of priapism is estimated at approximately 0.5 to 1.5 cases per 100,000 men annually, making it an uncommon but clinically significant condition [3].

Drug-induced priapism accounts for approximately one-third of all cases, with psychotropic medications being among the most frequently implicated agents [1,4]. Trazodone, a serotonin antagonist and reuptake inhibitor (SARI), is commonly prescribed for depression and insomnia but has been associated with priapism since its early clinical use [5]. The proposed mechanism involves alpha-adrenergic blockade, which leads to impaired venous outflow from the corpora cavernosa [1]. Despite being well-documented, trazodone-induced priapism remains underrecognized, especially when prescribed for non-psychiatric indications such as insomnia [6]. Early identification and structured management are crucial to avoid surgical intervention and preserve erectile function [2,7].

We present a case of ischemic priapism in a young male patient induced by trazodone, successfully managed with conservative therapy alone. This case highlights the importance of recognizing priapism as a potential adverse effect of trazodone, ensuring timely intervention, and avoiding unnecessary surgical morbidity [5,6].

## Case presentation

A 29-year-old man was admitted to the urology department with a painful penile erection lasting approximately 10 hours. The patient denied trauma, hematologic disorders, or use of phosphodiesterase inhibitors. He had no chronic diseases and was not on any medications except trazodone (Trittico 150 mg daily), which had recently been prescribed by a psychiatrist for insomnia [5]. According to the patient, trazodone therapy had started a few weeks before symptom onset, but he was unable to provide the exact timing, making a temporal relationship likely but imprecise.

On admission, physical examination revealed rigid corpora cavernosa with a soft glans and corpus spongiosum. The patient reported increasing pain and no resolution despite rest and cold compresses. Due to the emergent nature of management, ultrasound images were not stored; however, Doppler ultrasound confirmed absent cavernosal flow, consistent with ischemic priapism [1,2]. Cavernosal blood gas analysis showed pH 6.96, pCO₂ 99.8 mmHg, pO₂ 16.1 mmHg, HCO₃⁻ 22.9 mmol/L, base excess −8.9, SO₂ 19%, and lactate 13.8 mmol/L, confirming venous blood characteristics and severe local hypoxia (Table 1) [3].

**Table 1:** Cavernosal blood gas analysis These results confirm that the trapped cavernosal blood is severely hypoxic and acidotic, which is typical for ischemic (low-flow) priapism. mmol/L = millimoles per liter; mmHg = millimeters of mercury

Parameter	Abbreviation	Patient result	Reference range (venous blood)
pH	pH	6.96	7.31–7.41
Partial pressure of CO₂	pCO₂	99.8 mmHg	41–51 mmHg
Partial pressure of O₂	pO₂	16.1 mmHg	35–45 mmHg
Bicarbonate	HCO₃⁻	22.9 mmol/L	22–26 mmol/L
Base excess	BE	−8.9	−2.0 to +2.0
Oxygen saturation	SO₂	19%	60–85%
Lactate	Lactate	13.8 mmol/L	0.5–2.2 mmol/L

Bilateral cavernosal puncture was performed using two 20G cannulas near the glans penis. Mild sedation with midazolam (Dormicum 7.5 mg orally) and local anesthesia were used during the procedure. Dark, viscous blood was aspirated. Cavernosal irrigation with saline was initiated but was only partially effective due to intraluminal clot formation, likely related to prolonged priapism. Two ampules of ephedrine (25 mg each, diluted in 10 ml of 0.9% NaCl) were administered intracavernosally with partial detumescence. The patient received subcutaneous therapeutic-dose low-molecular-weight heparin (LMWH) (1 mg/kg), and this was repeated off-label after two hours. Although the use of LMWH is not routinely recommended in the current American Urological Association (AUA) or European Association of Urology (EAU) guidelines, it was employed in this case based on institutional protocol to minimize the risk of thrombotic complications related to prolonged vascular stasis. Prior studies have noted the potential role of anticoagulation in complex or prolonged priapism cases [6]. Given progressive improvement, the decision was made to avoid surgical shunting due to the associated risk of erectile dysfunction [6,7]. Adjunctive treatment included local application of ice packs for approximately five hours. Complete resolution of priapism was achieved. No recurrence was observed during 24 hours of hospitalization. The patient was discharged in good general condition with the following recommendations: continuation of anticoagulation therapy (Clexane 80 mg s.c. twice daily for five days), discontinuation of trazodone [5], referral to a psychiatric clinic for medication reassessment, and outpatient urology follow-up. He was advised to avoid sexual activity for two weeks and to present to the emergency department in case of recurrence [4,5].

## Discussion

Ischemic priapism is a rare but serious urological emergency that requires prompt intervention to prevent irreversible erectile dysfunction [1,2]. According to the 2025 EAU guidelines, first-line management includes cavernosal aspiration, irrigation with saline, and intracavernosal sympathomimetic injection to restore venous outflow [2]. In this case, early conservative treatment following these principles led to complete detumescence without the need for surgical shunting [6].

Although trazodone-induced priapism is uncommon, it can occur even at therapeutic doses, especially when prescribed for insomnia [4,5]. Discontinuation of the causative drug is essential, as recommended in current guidelines [5]. This case underlines the importance of recognizing trazodone as a potential trigger [4,8] and confirms that early, guideline-based conservative management can effectively resolve drug-induced ischemic priapism while preserving erectile function [3,9]. Recent advances in understanding molecular mechanisms may offer new preventive strategies [10].

## Conclusions

This case highlights that trazodone, even at therapeutic doses, can induce ischemic priapism requiring urgent urological intervention. Clinicians prescribing trazodone, particularly for off-label use such as insomnia, should be aware of this rare but serious adverse effect. Early recognition, prompt discontinuation of the offending agent, and adherence to evidence-based management guidelines from EAU (2025) can enable successful conservative treatment, potentially avoiding surgical intervention and long-term morbidity. In this case, ischemic priapism lasting over 10 hours was resolved with conservative measures alone, resulting in short-term preservation of erectile function, which underscores both the importance and effectiveness of timely, guideline-based care.
